# Apple RGL2a–JAZ4–MYC2 module orchestrates anthocyanin biosynthesis by regulating *MYB1* and *ERF3* expression

**DOI:** 10.1093/hr/uhag006

**Published:** 2026-01-06

**Authors:** Xiao-Wei Zhang, Baoyou Liu, Rui-Rui Xu, Lei Zhao, Yuepeng Han, Jian-Ping An

**Affiliations:** College of Horticulture Science and Engineering, Shandong Agricultural University, Taian 271018, China; Yantai Academy of Agricultural Sciences, Yantai 265599, China; College of Biology and Oceanography, Weifang University, Weifang 261061, China; State Key Laboratory of Plant Diversity and Specialty Crops, Wuhan Botanical Garden, Chinese Academy of Sciences, Wuhan 430074, China; State Key Laboratory of Plant Diversity and Specialty Crops, Wuhan Botanical Garden, Chinese Academy of Sciences, Wuhan 430074, China; State Key Laboratory of Plant Diversity and Specialty Crops, Wuhan Botanical Garden, Chinese Academy of Sciences, Wuhan 430074, China

Dear Editor,

Anthocyanins are important secondary metabolites widely distributed throughout plant organs. Their accumulation in stems and leaves enhances plant resistance to various abiotic stresses, while deposition in flowers and fruits facilitates pollinator and seed disperser attraction, thereby improving reproductive fitness. Anthocyanin accumulation serves as a key marker of fruit ripening, with anthocyanin-rich fruits being particularly valued for their superior antioxidant and free radical-scavenging capacities. Anthocyanins are biosynthesized through the phenylpropanoid pathway and are regulated by the MBW complex composed of MYELOBLASTOSIS (MYB), BASIC HELIX–LOOP–HELIX (bHLH), and WD40 proteins. In apple, MdMYB1, MdbHLH3/33, and TRANSPARENT TESTA GLABRA 1 (MdTTG1) are considered key positive regulatory components for anthocyanin biosynthesis. Plant hormones play an important role in the regulation of anthocyanin biosynthesis, and nearly all known hormones influence anthocyanin production. Although both jasmonic acid (JA) and gibberellin (GA) have been implicated in anthocyanin biosynthesis regulation, the molecular mechanisms underlying their interaction remain incompletely understood. In this study, we identified a novel JA signaling repressor, JASMONATE ZIM-DOMAIN 4 (MdJAZ4), which directly interacted with the GA signaling repressor RGA-LIKE 2a (MdRGL2a) to coordinately regulate anthocyanin biosynthesis in apple (*Malus* × *domestica*).

JAZ and DELLA proteins serve as core regulatory components in JA and GA signaling pathways, respectively. Although previous studies in apple have identified three key integrators including MdbHLH162, NAM, ATAF, AND CUC 72 (MdNAC72), and ZINC FINGER PROTEIN 7 (MdZFP7), that coordinate JA and GA signaling to regulate anthocyanin biosynthesis through their respective interactions with JAZ and DELLA proteins [1–3], whether JAZ and DELLA proteins interact directly remained unknown. Yeast two-hybrid (Y2H) assays revealed that three JAZ proteins including MdJAZ2, MdJAZ3, and MdJAZ4 could directly interact with the DELLA protein MdRGL2a (Fig. 1A). We focused on the MdJAZ4-MdRGL2a interaction as it showed the strongest growth phenotype in selective media. Then, the direct interaction between MdJAZ4 and MdRGL2a *in vitro* and *in vivo* was further confirmed through pull-down, bimolecular fluorescence complementation (BiFC), and co-immunoprecipitation (Co-IP) assays (Fig. 1B; Fig. S1). Exogenous methyl jasmonate (MeJA) treatment significantly reduced MdJAZ4 protein abundance, while pretreatment with the proteasome inhibitor MG132 blocked this degradation (Fig. 1C), demonstrating that MdJAZ4 is regulated by JA signaling through the 26S proteasome pathway. To investigate the biological function of MdJAZ4 in anthocyanin biosynthesis, we performed anthocyanin accumulation assays in apple fruits. *MdJAZ4* overexpression significantly suppressed anthocyanin accumulation and downregulated key anthocyanin biosynthesis-associated genes including *MdMYB1*, *CHALCONE SYNTHASE* (*MdCHS*), *CHALCONE ISOMERASE* (*MdCHI*), *flavanone 3-hydroxylase* (*MdF3H*), *DIHYDROFLAVONOL 4-REDUCTASE* (*MdDFR*), *anthocyanidin synthase* (*MdANS*), and *UDPGLUCOSE FLAVONOID 3-O-GLUCOSYLTRANSFERASE* (*MdUF3GT*), whereas *MdJAZ4* silencing enhanced anthocyanin production accompanied by upregulated expression of these genes (Fig. 1D–G). To further elucidate the role of MdJAZ4 in JA-induced anthocyanin biosynthesis, we treated wild-type, *MdJAZ4*-overexpressing, and *MdJAZ4*-suppressed apple callus with MeJA. Anthocyanin accumulation assays in apple callus showed that compared with wild-type and *MdJAZ4*-suppressed callus, the *MdJAZ4*-overexpressing callus exhibited markedly impaired MeJA-induced anthocyanin biosynthesis (Fig. 1H; Fig. S2). These results collectively demonstrate that MdJAZ4 acts as a negative regulator in JA-triggered anthocyanin accumulation.

**Figure 1:**
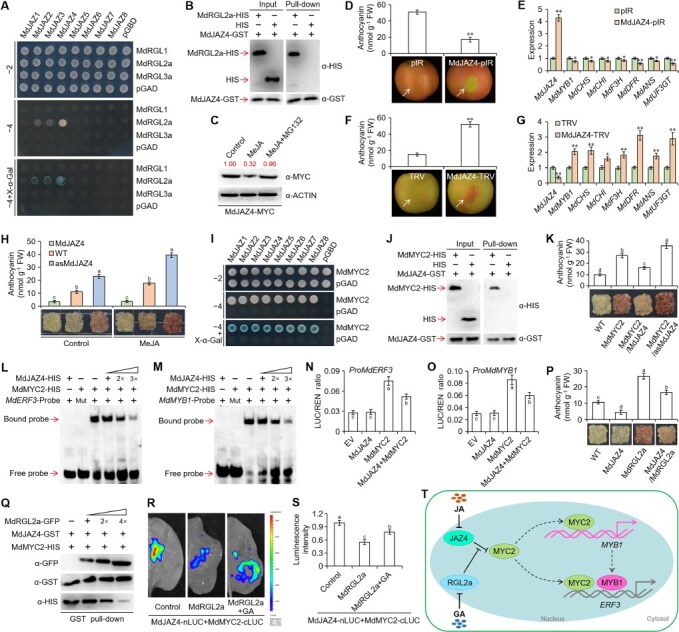
The MdRGL2a–MdJAZ4–MdMYC2 module regulates anthocyanin biosynthesis by modulating the expression of *MdERF3* and *MdMYB1*. (A) Y2H assay showing the interaction between JAZ and DELLA proteins. –2, medium lacking tryptophan and leucine. –4, medium lacking tryptophan, leucine, histidine, and adenine. (B) Pull-down assay showing the interaction between MdJAZ4 and MdRGL2a. (C) Analysis of MdJAZ4 protein abundance in *MdJAZ4*-overexpressing apple callus treated with 20 μM MeJA for 4 h, with untreated callus as control. MG132, 100 μM MG132 treatment for 0.5 h. (D) Anthocyanin accumulation phenotypes in *MdJAZ4*-overexpressed apple fruits (MdJAZ4-pIR) and empty vector controls (pIR) after 6 days of light treatment. pIR, IL60–1 + IL60–2; MdJAZ4-pIR, IL60–1 + MdJAZ4-IL60–2. The experiment was repeated three times with similar results, and each replicate included six to eight apple fruits. A representative image is shown here. The arrow indicates the injection site. (E) Quantitative reverse transcription PCR (qRT-PCR) showing the expression of *MdJAZ4* and anthocyanin biosynthesis-associated genes in apple fruits shown in D. The pIR value of each gene was normalized to 1. qPCR data were presented as means ± SD of three biological replicates (*n* = 3), with *MdACTIN* serving as the internal control. (F) Anthocyanin accumulation phenotypes in *MdJAZ4*-silenced apple fruits (MdJAZ4-TRV) and empty vector controls (TRV) after 3 days of light treatment. TRV, TRV1 + TRV2; MdJAZ4-TRV, TRV1 + MdJAZ4-TRV2. The experiment was repeated three times with similar results, and each replicate included six to eight apple fruits. A representative image was shown here. The arrow indicated the injection site. (G) qRT-PCR showing the expression of *MdJAZ4* and anthocyanin biosynthesis-associated genes in apple fruits shown in F. The TRV value of each gene was normalized to 1. qPCR data were presented as means ± SD of three biological replicates (n = 3), with *MdACTIN* serving as the internal control. (H) Anthocyanin accumulation phenotypes in *MdJAZ4* transgenic apple callus (MdJAZ4, overexpression of *MdJAZ4*; asMdJAZ4, suppression of *MdJAZ4*) and wild-type controls (WT) treated with 20 μM MeJA for 10 days, with untreated callus as controls. Apple callus was exposed to light for 10 days. The experiment was repeated three times with similar results, and each replicate included three to five callus masses. A representative image was shown here. (I) Y2H assay showing the interaction between MdMYC2 and JAZ proteins. (J) Pull-down assay showing the interaction between MdMYC2 and MdJAZ4. (K) Anthocyanin accumulation phenotypes in *MdJAZ4* and *MdMYC2* co-expressed apple callus after 10 days of light treatment. MdMYC2, overexpression of *MdMYC2*; MdMYC2/MdJAZ4, overexpression of *MdJAZ4* in *MdMYC2*-overexpressing background; MdMYC2/asMdJAZ4, suppression of *MdJAZ4* in *MdMYC2*-overexpressing background. The experiment was repeated three times with similar results, and each replicate included three to five callus masses. A representative image was shown here. (L, M) EMSA showing the effect of MdJAZ4 on MdMYC2 binding to the *MdERF3* and *MdMYB1* promoters. (N, O) Dual luciferase assay showing the effect of MdJAZ4 on MdMYC2 transcription activation of the *MdERF3* and *MdMYB1* promoters. (P) Anthocyanin accumulation phenotypes in *MdRGL2a* and *MdJAZ4* co-expressed apple callus after 10 days of light treatment. MdJAZ4, overexpression of *MdJAZ4*; MdRGL2a, overexpression of *MdRGL2a*; MdJAZ4/MdRGL2a, overexpression of *MdRGL2a* in *MdJAZ4*-overexpressing background. The experiment was repeated three times with similar results, and each replicate included three to five callus masses. A representative image was shown here. (Q) Competitive binding assay showing the effect of MdRGL2a on the MdJAZ4-MdMYC2 interaction. (R, S) Luciferase complementation imaging assay. The value of the empty vector control was normalized to 1. GA, leaves were sprayed with 10 μM GA_3_. The experiment was repeated three times with similar results, and each replicate included three to four *Nicotiana benthamiana* leaves. A representative image was shown here. (T) Model of the MdRGL2a–MdJAZ4–MdMYC2 module regulating anthocyanin biosynthesis in apple. Error bars represent SDs. Different lowercase letters indicate significant differences at *P* < 0.05 based on one-way ANOVA followed by Tukey’s test. Asterisks indicate significant differences according to *t*-tests. ^*^P < 0.05; ^**^P < 0.01.

As JAZ proteins lack transcriptional regulatory activity, they typically exert their regulatory functions by modulating co-regulators such as MYELOCYTOMATOSIS PROTEIN 2 (MYC2). Y2H assays revealed extensive interactions between MdMYC2 and multiple MdJAZ proteins, including MdJAZ4 (Fig. 1I), and the interaction between MdJAZ4 and MdMYC2 was further validated by pull-down, BiFC, and Co-IP assays (Fig. 1J; Fig. S3). Anthocyanin accumulation assays in apple callus demonstrated that *MdJAZ4* overexpression attenuated MdMYC2-promoted anthocyanin biosynthesis, whereas inhibiting *MdJAZ4* further promoted anthocyanin biosynthesis promoted by MdMYC2 (Fig. 1K; Fig. S4), suggesting the antagonistic role of MdJAZ4 in the regulation of MdMYC2-modulated anthocyanin biosynthesis. While previous studies have reported that MdMYC2 binds to and activates the promoter of *ETHYLENE RESPONSIVE FACTOR 3* (*MdERF3*), a positive regulator of anthocyanin biosynthesis [4], it remained unclear whether MdMYC2 directly activates *MdMYB1*. Through electrophoretic mobility shift assay (EMSA) and chromatin immunoprecipitation (ChIP)-polymerase chain reaction (PCR) assays, we confirmed that MdMYC2 directly bound to the *MdMYB1* promoter (Fig. S5A–D; Table S1). And dual-luciferase assays revealed that MdMYC2 trans-activated the *MdMYB1* promoter (Fig. S5E). Given the observed MdJAZ4–MdMYC2 interaction and MdMYC2’s transcriptional activation of both *MdERF3* and *MdMYB1* promoters, we investigated whether MdJAZ4 affects MdMYC2-mediated transcriptional regulation. EMSAs showed that while MdJAZ4 could not directly bind to *MdERF3* or *MdMYB1* promoters, it significantly attenuated MdMYC2’s binding affinity (Fig. 1L and M). This inhibitory effect was further supported by dual-luciferase assays showing MdJAZ4-mediated reduction of MdMYC2’s trans-activation capacity (Fig. 1N and O). Collectively, these findings demonstrate that MdJAZ4 negatively regulates MdMYC2-promoted anthocyanin biosynthesis by impairing MdMYC2’s transcriptional activation of both *MdERF3* and *MdMYB1*.

Subsequently, we investigated how MdRGL2a influences MdJAZ4’s regulatory function. Anthocyanin accumulation assays in apple callus demonstrated that *MdRGL2a* overexpression substantially alleviated MdJAZ4-mediated suppression of anthocyanin biosynthesis (Fig. 1P; Fig. S6). We hypothesized that MdRGL2a might interfere with the MdJAZ4–MdMYC2 interaction. Competitive binding assays revealed a dose-dependent weakening of MdJAZ4–MdMYC2 interaction strength when supplemented with increasing amounts of GFP-tagged MdRGL2a protein precipitated from *MdRGL2a*-overexpressing callus (Fig. 1Q). This dissociation effect was corroborated by luciferase complementation imaging assays, which consistently showed MdRGL2a’s ability to disrupt MdJAZ4–MdMYC2 interaction (Fig. 1R and S). Interestingly, GA treatment partially counteracted MdRGL2a’s inhibitory effect on MdJAZ4–MdMYC2 interaction (Fig. 1R and S), likely due to GA-induced degradation of MdRGL2a protein.

A regulatory model was proposed to depict how the MdRGL2a–MdJAZ4–MdMYC2 module coordinates anthocyanin biosynthesis through regulation of *MdMYB1* and *MdERF3* expressions (Fig. 1T). MdMYC2 promotes anthocyanin production via dual safeguard mechanisms: direct transcriptional activation of both *MdMYB1* and *MdERF3*, with the positive regulation of MdMYB1 on the *MdERF3* promoter having been previously established [5]. The JA signaling repressor MdJAZ4 attenuates MdMYC2-driven anthocyanin biosynthesis by impairing MdMYC2’s transcriptional activation of both target genes. Conversely, the GA signaling repressor MdRGL2a preserves MdMYC2 regulatory function by physically sequestering the MdJAZ4–MdMYC2 complex. Our findings not only elucidate a novel MdJAZ4–MdMYC2–*MdMYB1*/*MdERF3* pathway governing anthocyanin biosynthesis, but also reveal MdRGL2a–MdJAZ4 interaction as a molecular nexus integrating GA and JA signaling. This work establishes a new framework for investigating hormonal cross-talk in anthocyanin regulation, providing a paradigm for understanding phytohormone interplay in secondary metabolism.

## Data Availability

All data are available within the manuscript and the supporting materials in the figshare database (https://doi.org/10.6084/m9.figshare.30827474).
